# Patterns of anticoagulation therapy in atrial fibrillation: results from a large real-life single-center registry

**DOI:** 10.3325/cmj.2020.61.440

**Published:** 2020-10

**Authors:** Ivana Jurin, Marko Lucijanić, Zrinka Šakić, Vanja Hulak Karlak, Armin Atić, Ana Magličić, Boris Starčević, Irzal Hadžibegović

**Affiliations:** 1 Department for Cardiovascular Diseases, University Hospital Dubrava, Zagreb, Croatia; 2 Department of Hematology, University Hospital Dubrava, Zagreb, Croatia; 3 Health Center Zagreb East, Zagreb, Croatia; 4 Emergency Department, University Hospital Dubrava, Zagreb, Croatia; 5 Department of Oncology and Radiotherapy, University Hospital Center Zagreb, Zagreb, Croatia; 6 Faculty of Dental Medicine and Health Care, Josip Juraj Strossmayer University, Osijek, Croatia

## Abstract

**Aim:**

To investigate the differences in the characteristics and clinical outcomes of recently diagnosed patients with atrial fibrillation (AF) receiving different types of anticoagulants in a real-life setting.

**Methods:**

We retrospectively analyzed the charts of 1000 consecutive patients with non-valvular AF diagnosed and referred for hospitalization at our institution from 2013 to 2018.

**Results:**

Over the observed period, the frequency of direct oral anticoagulation (DOAC) therapy use significantly increased (*P* = 0.002). Patients receiving warfarin had more unfavorable thromboembolic and bleeding risk factors than patients receiving DOAC. Predetermined stroke and major bleeding risks were similarly distributed among the dabigatran, rivaroxaban, and apixaban groups. Patients receiving warfarin had shorter time-to-major bleeding (TTB), time to thrombosis (TTT), and overall survival (OS) than patients receiving DOACs. After adjustment for factors unbalanced at baseline, the warfarin group showed significantly shorter OS (hazard ratio 2.27, 95% confidence interval 1.44-3.57, *P* < 0.001], while TTB and TTT did not significantly differ between the groups. Only 37% of patients on warfarin had optimal dosing control, and they did not differ significantly in TTB, TTT, and OS from patients on DOACs.

**Conclusion:**

Warfarin and DOACs are administered to different target populations, possibly due to socio-economic reasons. Patients receiving warfarin rarely obtain optimal dosing control, and experience significantly shorter survival compared with patients receiving DOACs.

Atrial fibrillation (AF) is the most common arrhythmia, resulting in a 5-fold increased risk of stroke and non-cerebral systemic embolism, and imposing a major morbidity and mortality burden on elderly patients (1).

The stroke risk has been historically managed with vitamin K antagonists (VKA), such as warfarin, but the narrow therapeutic range and the requirement for frequent monitoring and dose adjustments have invoked the need for new therapeutic options (2). In 2011 and 2012, direct oral anticoagulation (DOACs) drugs were introduced as alternatives to warfarin for stroke prophylaxis in AF, and have since shown at least non-inferior efficacy and safety compared with warfarin in large randomized controlled trials (3-5). Due to fewer drug and food interactions, no need for frequent laboratory monitoring, and safety confirmed in large randomized clinical trials, DOACs usage has increased over the years. The penetration of DOACs differs across different health care systems and economic settings, and largely depends on reimbursement policies. In Croatia, DOACs are currently only partially (50%) reimbursed by the national health care provider (Croatian Institute for Health Insurance) for patients older than 65 years with an increased risk for thromboembolic event, whereas warfarin therapy and outpatient laboratory control of optimal warfarin dosing are fully reimbursed.

Our knowledge on optimal therapeutic strategies in thromboembolic protection in non-valvular AF in the “real world” settings is still limited. Randomized trials have enrolled patients with almost optimal therapeutic range of warfarin compared with DOACs, and no randomized controlled study has performed direct pairwise comparisons of safety and efficacy of different DOACs. Recently, some data have been published on real-life quality of warfarin therapy and DOAC penetration in South-Eastern Europe (6). However, there are no data on significant adverse events and changes of anticoagulation treatment strategy during long term follow-up in Croatia. Therefore, we aimed to investigate the characteristics and important clinical outcomes in a real-life group of AF patients newly exposed to different types and doses of anticoagulant drugs as a part of an initial strategy for stroke/systemic embolism risk reduction.

## Patients and methods

This retrospective study enrolled 1000 consecutive patients diagnosed with non-valvular AF and referred for hospital treatment in our institution from 2013 to 2018. The registry did not include patients with absolute indications for VKA, prosthetic mechanical valves, and moderate or severe mitral stenosis. The year 2013 was selected as a starting point for data collection because in that year DOACs became widely available and partially reimbursed in Croatia. Patients were included in the registry if they had not been previously exposed to any anticoagulation drug and if AF was recently diagnosed. Most patients received either DOAC or warfarin as initial part of stroke/systemic embolism risk reduction management and received other appropriate therapies according to guidelines. Demographic, clinical, and therapy data were collected at the start of follow-up from the patients’ electronic charts and by telephone visits.

Standard demographic, clinical, and echocardiographic data were collected, and additional risk scores were calculated. CHA2DS2-VASC score was calculated as follows: 1 point for chronic heart failure, 1 point for arterial hypertension, 1 point for age >65 years, 2 points for age >75 years, 1 point for diabetes mellitus, 2 points for history of stroke, TIA, or thromboembolism, 1 point for vascular disease, and 1 point for female sex. HAS-BLED score was calculated as follows: 1 point for arterial hypertension, 1 point for abnormal liver and liver function (each), 1 point for previous stroke, 1 point for previous bleeding, 1 point for labile INR, 1 point for age >65 years, 1 point for prior alcohol or drugs usage, and 1 point for medication predisposing to bleeding. LADS score included data on left atrial diameter, age, and diagnosis of stroke with a 6-point scoring system (1 or 2 points for each variable) based on these variables (7). HATCH score was calculated with either 1 or 2 points for each clinical category (hypertension – 1 point, age >75 years – 1 point, transient ischemic attack or stroke – 2 points, chronic obstructive pulmonary disease – 1 point, and heart failure – 2 points). The renal function was estimated with Cockcroft-Gault equation and expressed as creatinine clearance (CrCl) in mL/min/1.73m^2^.

After the enrollment, patients were followed up by accessing data from hospital information system and by telephone visits. Telephone visits were performed for all patients every 6 months in order to collect information on endpoints, therapy changes/switches, and optimal dosing. The last telephone visits were performed in February 2020. We evaluated optimal dosing for warfarin using at least 10 standardized international ratio (INR) values obtained at the last telephone visit and calculated it as the percentage of time in therapeutic range (TTR). INR between 2 and 3 was considered as optimal therapeutic range. At least 70% or more of the measured values in that range were considered as optimal dosing at the time of the last telephone visit, confirming that the patient is regularly taking warfarin (TTR≥70%, ie, 7/10 INR values between 2 and 3). Most of the patients had 10 values measured during a period of at least 10 months. If the patient experienced therapy switch or discontinuation before measuring at least 10 INR values, TTR was calculated from any available INR measurements at the time of the telephone visit. Optimal dosing for DOACs was assessed only at baseline, after the introduction of the first DOAC, using relevant demographic and clinical characteristics (age, body weight, and renal function) according to the DOAC dosing charts provided by the manufacturers. Patients with missing data on events during the follow-up were not included in the analyses, whereas data on therapy changes/switches, and optimal dosing were available in 92% and 96% of patients, respectively. The study was approved by the Institutional Review board of University Hospital Dubrava, Zagreb, Croatia.

### Statistical analysis

The normality of distribution of numerical variables was assessed with the Shapiro-Wilk test. Numerical variables are presented as median and interquartile range, and the significance of differences between the groups was assessed with the Kruskal-Wallis one-way analysis of variance and *post-hoc* Conover test. Categorical variables are presented as frequencies and percentages, and the significance of differences between the groups was assessed with the χ^2^ test. The trend of increase in DOAC use over time was tested using the χ^2^ test for trend. Survival analyses were conducted with the Kaplan-Meier method. Survival curves were univariately compared using the Mantel-Cox log-rank test. Multivariate regression analyses were performed using the Cox regression only for patients receiving warfarin (in general) or DOACs (dabigatran, rivaroxaban, or apixaban). All variables that differed significantly (Table 1) were included in the multivariate Cox regression analyses, except the variables included in the clinical scores in order to avoid collinearity. The final model was selected through a stepwise procedure with the “entry” and “stay” criterion of *P* ≤ 0.10. The final model for every endpoint was adjusted for the index year to avoid time trend bias. The endpoints were defined as follows: overall survival (OS) as the time from inclusion to death of any cause; time to thrombosis (TTT) as the time from inclusion to the first stroke/systemic embolism; time to bleeding (TTB) as the time from inclusion to the first major bleeding defined according to the International Society on Thrombosis and Hemostasis (8). In the case of any permanent therapy discontinuation not related to the patient’s death, or initial therapy switch before any of the endpoints, patients were censored. Unadjusted hazard ratio (HR) for TTB, TTT, and OS was calculated only for patients treated with oral anticoagulation comparing all patients receiving warfarin with the remaining patients receiving dabigatran, rivaroxaban, or apixaban at baseline. The patients who were not initially treated with oral anticoagulation therapy were not included in the HR analysis or multivariate Cox regression analyses. However, they were also followed for the assessment of the three defined endpoints (in an “intention to treat fashion”), and their event rate was expressed using Kaplan-Meier method. In addition, in the analysis of event rates using Kaplan-Meier method, patients treated with warfarin were divided into two groups: patients with TTR≥70% and patients with TTR<70%. Because of a low number of patients receiving suboptimal DOAC doses, DOAC patients were not additionally subgrouped regarding optimal dosing. The level of statistical significance was set at *P* < 0.05. Bonferroni correction for multiple simultaneous comparisons was used where appropriate. The analysis was performed with the IBM SPSS software, version 19 (IBM, Armonk, NY, USA).

**Table 1:** Demographic and clinical characteristics in regard to first-choice anticoagulation therapy in 1000 patients with AF*

Variable; median (range) or number (%)	Anticoagulation therapy					P
	none N = 141	warfarin N = 461	dabigatran N = 208	rivaroxaban N = 97	apixaban N = 93	
AF at presentation						
paroxysmal	114 (81)	171 (37)	118 (57)	45 (46)	49 (53)	<0.001
persistent	8 (6)	80 (17)	32 (15)	18 (19)	14 (15)	
permanent	19 (13)	210 (46)	58 (28)	34 (35)	30 (32)	
Age, median	62 (21-91)	73 (31-93)	70 (30-90)	70 (32-93)	72 (44-89)	<0.001
Male sex	86 (61)	226 (49)	119 (57)	48 (50)	45 (48)	0.058
BMI	26 (18-44)	28 (17-45)	27 (21-42)	28 (18-46)	28 (22-42)	<0.001
CrCl, mL/min/1.73m^2^	75 (23-143)	61 (13-137)	67 (26-144)	63 (8-190)	64 (30-139)	<0.001
LDL-C, mmol/L	3.1 (1.1-6.2)	2.9 (0.4-6.6)	2.9 (0.9-6.5)	3.0 (1.2-6.5)	3.0 (0.4-6.5)	<0.001
Hypertension	85 (60)	396 (86)	170 (81)	80 (83)	77 (83)	<0.001
Diabetes mellitus	16 (11)	107 (23)	44 (21)	19 (20)	19 (20)	0.050
Active tobacco use	31 (22)	71 (15)	34 (16)	19 (20)	15 (16)	0.421
CAD	13 (9)	90 (20)	27 (13)	10 (10)	10 (10)	0.005
Stroke/TIA	15 (11)	51 (11)	20 (10)	5 (5)	7 (8)	0.466
COPD	5 (4)	49 (11)	16 (8)	9 (9)	6 (7)	0.096
Malignancy	4 (3)	22 (5)	8 (4)	2 (2)	4 (4)	0.700
CHA2DS2Vasc score	2 (0-8)	4 (0-9)	3 (0-9)	3 (0-8)	3 (0-6)	<0.001
HAS-BLED score	1 (0-7)	2 (0-5)	2 (0-4)	2 (0-6)	2 (0-4)	<0.001
HATCH score	1 (0-6)	2 (0-7)	1 (0-7)	2 (0-6)	2 (0-6)	<0.001
LADS score	3 (0-6)	4 (0-6)	3 (0-6)	3 (1-5)	3 (0-6)	<0.001
LA diameter, cm	4.0 (2.7-6.0)	4.5 (2.9-7.7)	4.4 (2.7-6.6)	4.4 (3.0-6.2)	4.4 (2.3-6.9)	<0.001
LVEF, %	62 (30-78)	55 (17-79)	58 (20-77)	55 (30-77)	57 (15-78)	<0.001
Anticoagulation therapy change	53 (38)	103 (22)	21 (10)	11 (11)	7 (8)	<0.001
Optimal dosing	NA	171 (37)	192 (92)	92 (95)	91 (98)	<0.001
Bleeding event	2 (1)	40 (9)	4 (2)	11 (11)	2 (2)	<0.001
Thromboembolic event	20 (14)	45 (10)	8 (4)	2 (2)	0	<0.001
Death	31 (22)	146 (32)	13 (6)	11 (11)	6 (6)	<0.001

*AF – atrial fibrillation; BMI – body mass index; CrCl – creatinine clearance; LDL – C-low density lipoprotein cholesterol; CAD – coronary artery disease; TIA – transitory ischemic attack; COPD – chronic obstructive pulmonary disease; LA – left atrium; LVEF – left ventricular ejection fraction.

## Results

### Overall characteristics, risk factors, and initial anticoagulation strategies

We analyzed the data of 1000 patients with AF. The median age was 72 years (range 21 to 93 years). There was a similar proportion of male (524/1000, 52%) and female patients (476/1000, 48%). The median CHA2DS2VASC score was 3 points (range 0-9), with 847/1000 (85%) of patients having a substantial risk of stroke defined as score of ≥2 points. Median HAS-BLED score was 2 points (range 0-7), with 291/1000 (28%) of patients having a high risk of major bleeding defined as score of ≥3 points.

A majority of patients (859/1000, 86%) received oral anticoagulation after AF diagnosis, whereas the remaining 141/1000 (14%) patients did not receive any type of long term anticoagulation therapy, or were administered only long-term antiplatelet drugs, mostly acetylsalicylic acid, by discretion of a designated physician or if they rejected recommended anticoagulation therapy. As expected, these two subgroups significantly differed in predetermined stroke risk (*P* < 0.001; median CHA2DS2VASC scores 3 vs 2 for anticoagulated and no-anticoagulation therapy or ASA patients, respectively), and in predetermined risk for major bleeding (*P* < 0.001, median HAS-BLED scores 2 vs 1, respectively).

### Characteristics of anticoagulated patients

The median age of 859 patients receiving oral anticoagulation was 72 years (range 31-93 years). There were 438/859 (51%) male patients. Most patients were initially anticoagulated with warfarin [461/859 (54%)], followed by dabigatran [208/859 (24%)], rivaroxaban [97/859 (11%)], and apixaban [93/859 (11%)]. There was a significant trend of increase in the frequency of DOAC use over time, and the penetration of DOACs reached 62% by the end of 2018 (P for trend = 0.002). The increase in DOAC use resulted from an increased initial selection (from 33% in the index year 2013-2014 to 57% in the index year 2017-2018), but also from switching from warfarin or no oral anticoagulation strategy at baseline (Table 2). Changes in the initial anticoagulation strategy were significantly more frequent among patients who received no oral anticoagulant (38%) and among patients initially treated with warfarin (22%), with 97/461 (21%) patients switched from warfarin to any DOAC during the observed period (Table 1 and Table 2). Among warfarin-anticoagulated patients, optimal dosing with TTR of ≥70% during the last follow-up was recorded in 171/461 (37%) patients. Optimal doses of DOACs were used in >90% of patients, with no significant differences among different drug classes (Table 1).

**Table 2:** Patterns of anticoagulation therapy initiation or change in regard to the index years*

Anticoagulation therapy, No (%)	Index year			
	2013-2014	2015-2016	2017-2018	total
No OAC				
initial	67 (16)	47 (15)	27 (10)	141 (14)
warfarin stop	/	2	4	6
DOAC stop	/	2	2	4
Warfarin				
initial	210 (51)	164 (51)	87 (33)	461 (46)
switch from no OAC	2	3	2	7
switch from DOAC (any)	2	3	7	12
Dabigatran				
initial	65 (16)	65 (20)	78 (29)	208 (21)
switch from no OAC	2	9	12	23
switch from warfarin	2	5	26	33
switch from DOAC (other)	1	1	3	5
Rivaroxaban				
initial	25 (6)	26 (8)	46 (17)	97 (10)
switch from no OAC	2	4	7	13
switch from warfarin	/	4	30	34
switch from DOAC (other)	2	5	2	9
Apixaban				
initial	45 (11)	19 (6)	29 (11)	93 (9)
switch from no OAC	/	2	8	10
switch from warfarin	6	4	26	36
switch from DOAC (other)	2	1	10	13
Patients initially assigned to any strategy of anticoagulation	412 (100)	321 (100)	267 (100)	1000 (100)

*OAC – oral anticoagulation drug, DOAC – direct oral anticoagulation drug.

Patients who received no oral anticoagulation differed significantly in most demographic and clinical characteristics. As for anticoagulated patients, patients receiving warfarin were significantly older, were more likely to have lower CrCl, coronary artery disease, permanent AF, higher CHA2DS2VASC and HAS-BLED scores than DOAC receiving patients, showing that warfarin was administered to patients with more unfavorable clinical characteristics (data not shown, *P* < 0.001 for all comparisons; Table 1).

Among patients receiving DOAC, those receiving dabigatran were more likely to be male and to have lower BMI than those receiving rivaroxaban or apixaban (*P* < 0.05 for all comparisons; Table 1). Other clinical characteristics and predetermined stroke (CHA2DS2VASC, HATCH) and major bleeding risks (HAS-BLED, age, CrCl) were similarly distributed among these three groups.

### Clinical outcomes associated with different anticoagulation patterns

The median follow-up was 42 months. Endpoints selected for assessment were thrombosis (first documented thrombotic/thromboembolic event), bleeding (first documented bleeding event defined according to the International Society on Thrombosis and Hemostasis definitions), and death (overall survival).

**Figure 1:**
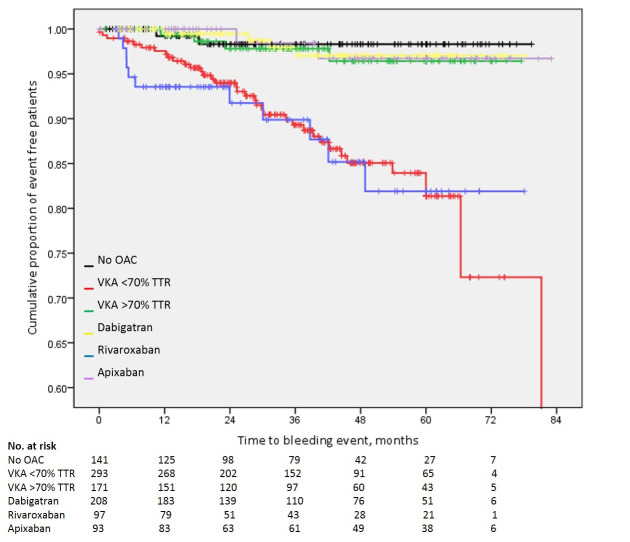
Time to bleeding event among atrial fibrillation patients with different initial anticoagulation strategies.

During the follow-up, 59 (5.9%) patients experienced a major bleeding event. The patients receiving no oral anticoagulation, warfarin with TTR of ≥70%, dabigatran, and apixaban experienced similar and significantly longer TTB than the patients receiving warfarin with TTR of <70% and patients receiving rivaroxaban (Mantel-Cox Log rank, *P* < 0.001, Figure 1). After adjusting for factors that significantly differed at baseline between warfarin and DOAC subsets (Table 1 and 3), an independent association of warfarin therapy to major bleeding in comparison with DOAC subsets was lost in the multivariate regression analysis (Table 3). In addition, higher BMI and HAS-BLED score, and lower CrCl were independently associated with bleeding events, whereas optimal dosing was independently associated with less bleeding. Adjusting for index year did not change the results of multivariate analysis (Table 3).

**Table 3:** Cox proportional hazard regression analysis of the impact of relevant clinical characteristics and choice of anticoagulation therapy on death, bleeding, and thrombotic events of patients receiving different anticoagulation therapy at baseline, adjusted for index year*

Variable*	Multivariate Cox regression, event, HR (CI)		
	death	bleeding event	thromboembolic event
Age	1.075 (1.048-1.103)†	1.020 (0.978-1.062)	1.002 (0.964-1.041)
CrCl	0.985 (0.976-0.994)†	0.983 (0.967-0.998)†	1.000 (0.985-1.083)
BMI	1.044 (1.007-1.082)†	1.075 (1.016-1.138)†	1.012 (0.945-1.083)
CHA2DS2-Vasc score	0.973 (0.851-1.112)	0.909 (0.715-1.155)	0.814 (0.630-1.051)
HAS-BLED score	1.101 (0.902-1.344)	1.982 (1.410-2.785)†	1.182 (0.818-1.710)
HATCH score	1.123 (0.981-1.285)	1.081 (0.862-1.356)	1.718 (1.354-2.181)†
LADS score	1.299 (1.101-1.532)†	1.079 (0.820-1.420)	1.281 (0.977-1.678)
Optimal dosing	0.553 (0.384-0.795)†	0.231 (0.109-0.492)†	0.198 (0.091-0.434)†
Warfarin vs DOAC	2.267 (1.441-3.567)†	0.574 (0.264-1.250)	2.232 (0.882-5.647)

*HR – hazard ratio; CI – confidence interval; CrCl – creatinine clearance; BMI – body mass index; DOAC – direct oral anticoagulant drug.

†Statistically significant independent association, *P* < 0.05.

**Figure 2:**
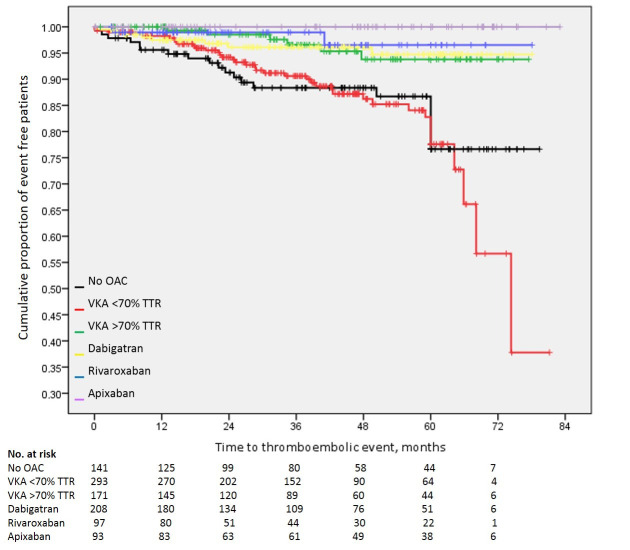
Time to thromboembolic event among atrial fibrillation patients with different initial anticoagulation strategies.

During the follow-up, 75 (7.5%) patients experienced a thrombotic event. There were 20 thrombotic events among 141 patients without oral anticoagulation therapy (14%) and 55 thrombotic events in 859 patients on oral anticoagulation therapy (6.4%). Patients receiving warfarin with TTR of ≥70%, dabigatran, rivaroxaban, or apixaban had significantly longer TTT than patients without oral anticoagulation and patients with TTR on warfarin of <70% (Mantel-Cox Log Rank, *P* < 0.001, Figure 2). Multivariate regression analysis, after adjusting for factors that significantly differed at baseline between the groups (Table 1 and 2), showed that the warfarin group had a HR for a thrombotic event of 2.23 (95% CI 0.88-5.65) compared with DOAC subgroups. After adjusting analyses for sex and BMI (unbalanced at baseline between DOAC subclasses), difference in TTT among three DOAC subclasses did not reach statistical significance. In addition, higher HATCH score was independently associated with thromboembolic events, whereas optimal dosing was independently protective against thrombotic events (Table 2). Adjusting for index year did not change the results of multivariate analysis (Table 3).

**Figure 3:**
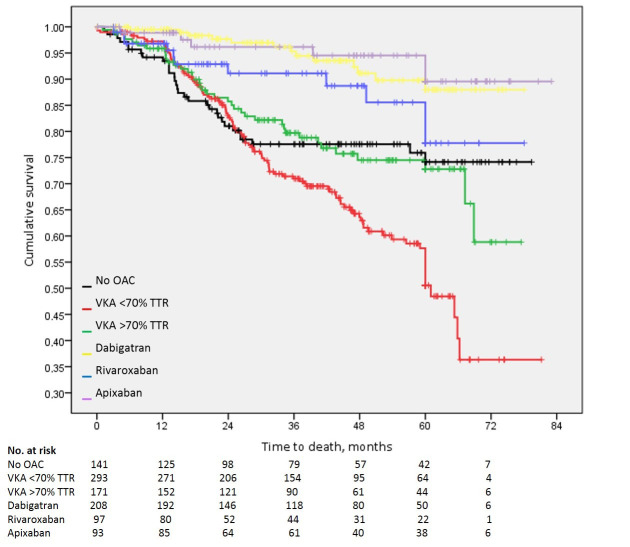
Overall survival of atrial fibrillation patients with different initial anticoagulation strategies.

During the follow-up, 207 (21%) patients died. There were 31 recorded deaths among 141 patients (22%) who received no anticoagulation therapy at baseline and 176 deaths among 859 (20%) patients who received anticoagulation therapy. OS significantly differed among subgroups of anticoagulated patients (*P* < 0.001) (Figure 3, Mantel-Cox Log Rank, *P* < 0.001). Patients receiving dabigatran, rivaroxaban, and apixaban had similar OS with superimposable survival curves (*P* = 0.916). However, every DOAC subgroup experienced superior survival in comparison to all warfarin-treated patients (unadjusted HR for dabigatran vs warfarin 0.16 with 95% CI (0.09-0.28), *P* < 0.001; HR for rivaroxaban vs warfarin 0.28 with 95% CI 0.14-0.54, *P* < 0.001; HR for apixaban vs warfarin 0.15 with 95% CI 0.06-0.35, *P* < 0.001). This finding persisted in the multivariate regression analysis after adjusting for factors that significantly differed at baseline between warfarin and DOAC subsets, showing warfarin therapy to be independently associated with shorter OS in comparison with DOACs (HR 2.27, 95% CI 1.44-3.57; Table 3). In addition, older age, lower CrCl, higher BMI, and higher LADS were also independently associated with shorter OS, whereas optimal dosing was associated with longer OS (Table 3). Adjusting for index year did not abrogate independent prognostic significance of optimal dosing, as well as warfarin therapy selection, independent of dosing quality or any other relevant factor.

## Discussion

To the best of our knowledge, this is the first study with a long-term follow-up of recently diagnosed AF patients receiving different anticoagulation strategies that assessed data on optimal therapy dosing and changes of the initial anticoagulation strategy in a real-life setting in Croatia. Our study demonstrated that patients with AF receiving different types of anticoagulants differed in baseline clinical characteristics but also experienced different risks of thrombosis, bleeding, and death. In other words, warfarin and DOACs in Croatia are most probably administered to different target populations, which is translated into significantly different clinical outcomes. We also showed that patients without optimal warfarin dosing experienced significantly more events and had the worst OS. Several recent reports suggest this trend in other countries as well, with patients receiving warfarin being older and having a more comorbidities (9,10). Up to date, no study representative of the Croatian health care system, assessed how these differences reflected on clinical outcomes. Recent BALKAN-AF survey (6), with only 6% patients from Croatia, demonstrated an overall low quality of VKA therapy, with 29.5% of patients being in therapeutic range ≥65% of time during the three months before the survey. A recent study from a southern Croatian county showed that the percentage of patients with AF on warfarin with TTR≥70% was only 23% (11). A randomized control study of community pharmacist interventions among Croatian elderly rural patients reported this proportion to be as low as 7.6% (12). Our study, conducted in an urban metropolitan area, also observed a rather low proportion of patients in adequate therapeutic range (37%) and again showed that proper therapeutic range for warfarin was hard to achieve in a real-life setting. The BALKAN-AF survey (6) also reported that patients with AF receiving DOACs more frequently had lower predetermined stroke and bleeding risks, which is in line with our results.

In our opinion, different patients’ characteristics associated with the use of different OACs could be attributed to the fact that DOACs are more expensive and are still only partially reimbursed by the Croatian Institute for Health Insurance. Therefore, among other possible reasons, warfarin is prescribed to patients who are not able to pay for DOACs, introducing a baseline socio-economic selection bias into investigated groups. This warrants further and more detailed investigations and structured decision-making algorithms, as it was already suggested in BALKAN-AF survey (6), as well as a continuous effort to make DOACs available to every patient who would benefit from their use, according to evidence-based medical practice. This also highlights the need to establish a functional registry of AF patients, as it was proposed earlier (13). We showed that DOAC use significantly increased during the follow-up, with DOACs representing more than 60% of newly introduced anticoagulants in 2018. We anticipate that the trend will continue as the prices of these agents decline, and as they become readily available for all patients groups with the indication for their use. In addition, with the relatively recent approval of reversal agents, the fear of prescribing DOACs is also expected to decline. Nevertheless, a substantial number of patients is still receiving warfarin as a first choice for thromboembolic protection in AF (almost 40% of newly introduced anticoagulants in 2018 in our study).

We demonstrated that optimal dosing was independently protective against unwanted events. Optimal warfarin dosing, although hard to achieve, involved similar bleeding and thrombotic risks as DOAC therapy. Patients initially treated with rivaroxaban experienced similar bleeding event rates as patients on warfarin, whereas DOACs with twice-daily dosing regimen (dabigatran or apixaban) and optimally titrated warfarin showed similar and very low bleeding risks. Since the three subgroups of patients receiving DOAC did not differ in bleeding risk scores, it seems that in our patients once-daily dosing regimen of DOACs might be associated with an increased bleeding risk. Head-to-head comparisons of individual DOACS are scarce, which makes it practically impossible to recommend one DOAC over another, although there seems to be a trend of better safety with dabigatran and apixaban (14,15). However, dabigatran seems to be associated with more pharmacokinetic concerns (16-18), and its drug concentration might be affected by obesity and other factors to a greater extent than that of other DOACs (19), consequently hindering its efficacy in a real-life setting. Our results showed that obesity was associated with an increased risk of bleeding and shorter OS, independent from the index year, anticoagulation strategy, or optimal dosing. Our results do not provide indisputable evidence for differences in efficacy/safety between the two DOAC subclasses (both factor Xa inhibitors and thrombin inhibitor) and we do not consider these to be clinically evident at the moment, but our data do hint that these differences might exist. What the present study showed more clearly is that the patients receiving either of the two DOAC subclasses had superior survival than warfarin-treated patients in general – which reflects the real-life prescription strategies in AF. Not only that in large randomized controlled trials, DOACs demonstrated at least non-inferior efficacy and safety compared with warfarin (3-5), but an increasing number of articles and meta-analyses (20-22) report that DOACs confer greater clinical benefit. Therefore, patient selection, but also better regulation of optimal drug concentration or direct effects of particular drugs, might be responsible for better outcomes. It should be noted that higher bleeding and thrombotic risk associated with warfarin diminished in the multivariate analysis, in contrast to overall mortality risks, which persisted after adjusting analyses for multiple factors unbalanced at baseline and index year. Even though bleeding risk might be of smallest magnitude, patients on DOACs who experience bleeding might require lower resource consumption and have a better short-term prognosis (23). However, this is still a subject of debate (24).

Finally, our study was also the first to enroll patients who were not anticoagulated after the AF diagnosis. One third of them experienced a change in initial no-OAC strategy to oral anticoagulation (mostly DOAC) during follow-up (due to aging, newly diagnosed risk factors etc). This subgroup of patients is often underreported in registries and prospective trials. Here, it consisted mostly of patients with more favorable risk profile: younger patients with paroxysmal AF and low thromboembolic risk, or extremely rarely, of very high-risk patients with comorbidities impeding optimal anticoagulation strategy. These patients, despite being significantly younger and had a more favorable risk profile, had thrombosis rates similar to patients with poorly controlled warfarin, and significantly worse OS than patients on DOACs. This calls for a more scrutinized approach in the treatment of younger patients with AF (paroxysmal or persistent) and lower thromboembolic and bleeding risk, and definitely warrants further study. These patients were not included in the multivariate analysis of the factors influencing outcomes because outcome analyses were directed exclusively to different initial anticoagulation strategies (warfarin vs DOACs).

The limitations of this study are single-center experience, retrospective study design, and insufficient statistical power for some of the presented analyses, mainly outcome analyses. Our method of TTR calculation, an important post-baseline parameter in patients treated with warfarin, was limited to relatively few INR measurements, preventing us from using linear interpolation or other more reliable methods. However, it again demonstrated relatively poor INR control in patients taking warfarin in our region (6). Also, multivariate analyses may have been biased due to a small number of recorded events among DOAC treated patients (worth noting – no observed thromboembolic events in the apixaban subgroup). Finally, our main observation, that older patients with higher thrombotic and bleeding receive warfarin more frequently probably because of socio-economic reasons, is speculative, because we were not able to objectively investigate the patients’ socioeconomic status.

Nevertheless, our real-life data on anticoagulation patterns in patients with AF reveal that DOACs and warfarin are administered to different target populations, which experience different clinical outcomes. Our findings, which need to be further evaluated, indicate the need for establishing a functional AF registry and for changing the current policies of DOAC reimbursement, or depending on future registry data and cost benefit analyses, real-life improvements in the optimization of warfarin dosing (introduction of widespread dedicated anticoagulation outpatient clinics or reimbursement for home INR monitoring devices). In conclusion, DOACs are increasingly used as anticoagulant drugs of choice in patients with AF and are currently the first choice anticoagulation strategy in more than 60% of patients with non-valvular AF. Our data suggest that different target populations receive warfarin and DOACs, possibly due to socio-economic reasons. Seemingly low-risk patients with AF should undergo a more scrutinized approach to therapy selection and diagnostic work-up. Twice-daily day dosing regimen of DOACs proved to be effective and very safe. Patients receiving warfarin in Croatia have difficulties in reaching optimal therapeutic effect and experience higher risks of thrombosis and bleeding, probably reflecting differences in patients’ characteristics and predetermined stroke/bleeding risk at baseline. However, significant differences in OS between warfarin and DOAC groups were not abrogated by any relevant factor unbalanced at baseline.

Competing interests ML is a statistical editor in the *Croatian Medical Journal*. To ensure that any possible conflict of interest relevant to the journal has been addressed, this article was reviewed according to best practice guidelines of international editorial organizations. All authors have completed the Unified Competing Interest form at www.icmje.org/coi_disclosure.pdf (available on request from the corresponding author) and declare: IH received personal fees from Boehringer Ingelheim, Pfizer, and Bayer unrelated to the submitted work; IJ received personal fees from Boehringer Ingelheim and Pfizer unrelated to the submitted work; BS received personal fees from Boehringer Ingelheim, Pfizer, and Bayer unrelated to the submitted work. They declare no support from any organization for the submitted work; no other financial relationships with any organizations that might have an interest in the submitted work in the previous 3 years; no other relationships or activities that could appear to have influenced the submitted work.
